# *Tigridiopalma exalata*, a new and endangered species of Melastomataceae from China

**DOI:** 10.3897/phytokeys.176.63619

**Published:** 2021-04-16

**Authors:** Si-Jin Zeng, Ye-Chun Xu, Gang-Tao Wang, Peng Jia, Da-Fang Cui

**Affiliations:** 1 College of Forestry and Landscape Architecture/Guangdong Key Laboratory for Innovative Development and Utilization of Forest Plant Germplasm, South China Agricultural University, Guangzhou 510642, Guangdong, China; 2 Key Laboratory of Plant Resources Conservation and Sustainable Utilization, South China Botanical Garden, Chinese Academy of Sciences, Guangzhou 510650, Guangdong, China; 3 Environmental Horticulture Research Institute/Guangdong Provincial Key Laboratory of Ornamental Plant Germplasm Innovation and Utilization, Guangdong Academy of Agricultural Sciences, Guangzhou 510640, Guangdong, China; 4 University of Chinese Academy of Sciences, Beijing 100049, China; 5 Guangzhou Institute of Forestry and Landscape Architecture, Guangzhou 510420, Guangdong, China

**Keywords:** Chinese melastomes, Guangdong, monospecific genus, new species, Sonerileae

## Abstract

A new species of the genus *Tigridiopalma*, formerly considered monotypic, is here described as *T. exalata* and illustrated based on molecular and morphological evidence. It is morphologically similar to *T. magnifica* in having a short stem, huge basal leaves, scorpioid cymes, and 5-merous flowers, but differs in having ribbed and pale yellow puberulent petioles, purple petals with a small white apical patch, connectives of longer stamens with a distinct dorsal short spur at their base, and wingless capsules. Due to the restricted distribution, small populations and horticultural potential of this new species, it should be categorized as an Endangered species (EN).

## Introduction

The genus *Tigridiopalma* C.Chen, with its sole species, *Tigridiopalma magnifica* C.Chen, is endemic to China (Chen 1984; Chen and Renner 2007; Lin and Xiong 2007). It is characterized by having a short and stoloniferous stem, huge leaves (up to 70 cm), scorpioid cymes, 5-merous flowers, 10 dimorphic and unequal stamens, and an ovary with a 5-lobed membranous crown at apex. The stamen connectives are decurrent, slightly spurred or forming a short spur, of which the bases are 2-tuberculate (Chen 1979, 1984; Chen and Renner 2007). *Tigridiopalma* was first placed in the tribe Sonerileae Triana by Chen (1984), and this placement was accepted by subsequent researchers (Renner 1993; Cellinese 1997; Cellinese 1999). Recent phylogenetic studies showed that *Tigridiopalma* belongs to Sonerileae, a major clade in the family although there is still uncertainty regarding the relationships of genera, where it is closely related to *Driessenia* Korth., *Heteroblemma* (Blume) Cámara-Leret, Ridd.-Num. & Veldkamp, *Medinilla* Gaudich., *Phyllagathis* Blume, *Scorpiothyrsus* H.L.Li and *Tashiroea* Matsum. ex T.Itô & Matsum. (Zeng et al. 2016; Zhou et al. 2019a, b).

**Figure 1.**
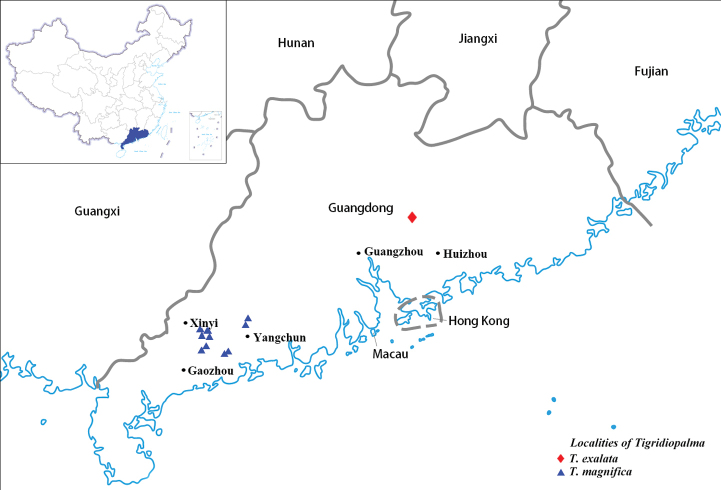
The distribution map of *Tigridiopalma*.

*Tigridiopalma magnifica* is only found in the Western Guangdong presently (Gaozhou, Xinyi and Yangchun, Fig. 1) (Chen and Renner 2007; Li et al. 2009; Ren et al. 2012). Due to its restricted distribution and small populations, it was categorized as an Endangered species (EN) in the latest version (2013) of the IUCN Red List of China and the Threatened Species List of China’s Higher Plants (Qin et al. 2017).

Here we describe a new species of *Tigridiopalma*, *T. exalata* S.Jin Zeng, Y.C.Xu & D.F.Cui, from Eastern Guangdong, China. It can be easily distinguished from *T. magnifica* by having ribbed and pale yellow puberulent petioles (vs. not ribbed and reddish hispid), purple petals with a small white apical patch (vs. dark red petals with a large white apical patch), connectives of longer stamens with a distinct dorsal short spur at the base (vs. indistinct) and wingless (vs. narrowly winged) capsules. We also provide a phylogenetic analysis confirming that the new species belongs to *Tigridiopalma*.

## Materials and methods

The morphological data collected for the species described here are based on living plants and specimens collected in the field. Voucher specimens were deposited at the herbaria CANT, IBSC, KUN and PE (acronyms according to Index Herbariorum in Thiers 2021).

We collected and sequenced the specimen (S.Jin Zeng 397) and then built a phylogenetic hypothesis with other sequences gathered from Genbank. Total genomic DNA was extracted from fresh material using a modified CTAB procedure (Smith et al. 1991). One nuclear DNA region (internal transcribed spacer, nrITS) and two plastid DNA markers (*ndhF* and *rpl16*) were used in this study following by Zeng et al. (2016).

Amplification and sequencing were performed according to Zeng et al. (2016). The primers used for Polymerase Chain Reactions (PCRs) are listed in Suppl. material 1: Table S1. PCRs were performed in a reaction mix (30 μL) containing total DNA (1 μL), primers (2 μL each), 2× MightyAmp Buffer (Ver.2) (15 μL), MightyAmp DNA polymerase (Takara Bio) (0.5 μL) and H_2_O (9.5 μL). The PCR program consisted of an initial 3 min pre-melt stage at 98 °C, followed by 38–42 cycles of 20–30 s at 98 °C (denaturation), 30 s at 45–58 °C, and 60–120 s at 68 °C, followed by a final 7 min extension at 68 °C. The PCR products were run on 1.5% agarose gels to check the quality of amplified DNA. Target products were excised from these gels, purified and sequenced by Invitrogen (Shanghai). Both forward and reverse sequences were edited and assembled with DNASTAR (http://www.dnastar.com/). DNA sequences were aligned in MEGA 7 (Kumar et al. 2016) using MUSCLE (Edgar 2004) and manually adjusted to account for obvious errors.

To determine the phylogenetic position of the new species in the Sonerileae clade, 45 species from 22 genera (including 2 species of *Tigridiopalma*) were used for molecular analyses (Suppl. material 1: Table S2). *Dissochaeta vacillans* Blume and *Pseudodissochaeta lanceata* Nayar were selected as outgroup taxa based on Zhou et al. (2019b). Maximum likelihood (ML) analysis was performed by RAxML-HPC2 on XSEDE (Stamatakis 2014) through the CIPRES portal (Miller et al. 2010) using GTRCAT model and 1000 bootstraps.

## Results

**Figure 2.**
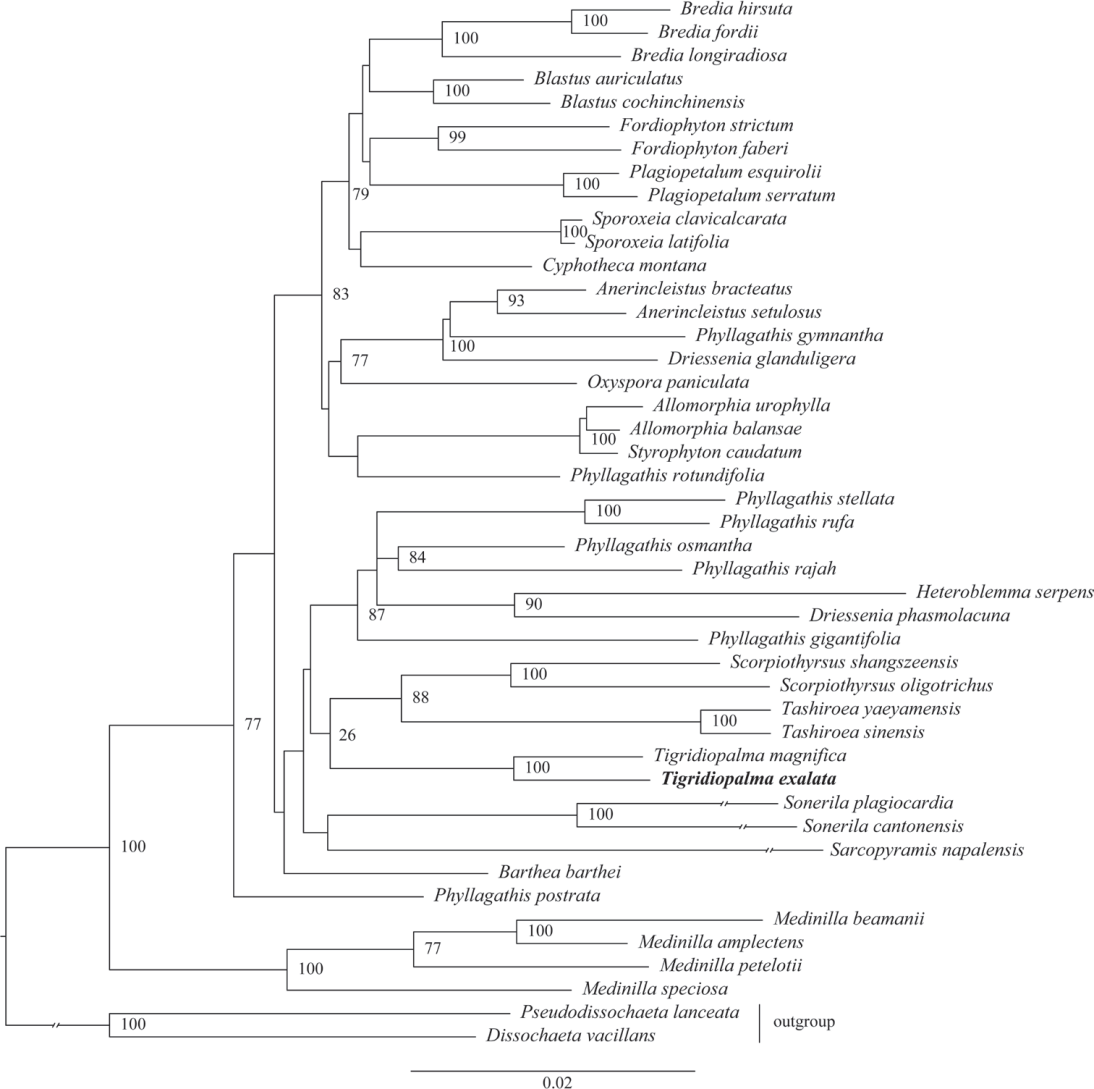
Phylogenetic relationships of Sonerileae based on combined nuclear ITS and two plastid makers (*ndhF*+*rpl16*). The maximum likelihood bootstrap values above 70 are shown at the nodes.

The aligned sequence matrix contained 2694 characters. Summary features of sampled sequences are summarised in Suppl. material 1: Table S3. The phylogenetic analysis indicated that the genus *Tigridiopalma* is recovered sister to *Scorpiothyrsus* plus *Tashiroea* clade (BS_ML_ 26), and the new species is sister to *Tigridiopalma magnifica* with strong support (BS_ML_ 100) (Fig. 2).

A detailed comparison of this new species and *T. magnifica* is shown in Table 1.

**Table 1.** Morphological comparisons of *Tigridiopalma exalata* and *T. magnifica*.

Items/species	*T. exalata*	*T. magnifica*
Petioles	abaxially ribbed, densely pale yellow puberulent trichomes	abaxially not ribbed, densely reddish hispid trichomes
Leaf blades	abaxially densely pale yellow puberulent trichomes on veins	abaxially densely reddish villous and puberulent trichomes on veins
Petals	purple with a small white apical patch	dark red with a large white apical patch
Connectives of longer stamens	basally with a distinct dorsal short spur	basally without a distinct dorsal spur
Capsules	wingless	narrowly winged

## Discussion

Zeng et al. (2016) first investigated the phylogenetic position of *Tigridiopalma* by using four makers (nrITS, *ndhF*, *rbcL* and *rpl16*) and found that it was close to *Sonerila* Roxb. and *Tashiroea* with moderate support (BS_ML_ 73). Subsequently, more taxa were added to reconstruct the phylogeny of Sonerileae with two markers (nrITS and *trnV*-*trnM* spacer), which indicated that *Tigridiopalma* was sister to *Medinilla* with weak support (BS_ML_ 49) (Zhou et al. 2019a). Subsequent phylogenomic analyses of Sonerileae using 171 plastid genomes showed that *Tigridiopalma* is sister to the clade consisting of *Driessenia*, *Heteroblemma*, *Medinilla* and some species of *Phyllagathis* with moderate support (BS_ML_ 71) (Zhou et al. 2019b). Due to the highly structurally conserved plastomes of Melastomataceae (Reginato et al. 2016; Zhou et al. 2019b), more nuclear DNA makers or SNPs are required to resolve the generic relationships of Sonerileae, including the closest relatives of *Tigridiopalma*.

### Taxonomic treatment

#### Tigridiopalma exalata

Taxon classificationPlantaeMyrtalesMelastomataceae

S.Jin Zeng, Y.C.Xu & D.F.Cui sp. nov.

DE3FD816-9380-5E53-8B86-8D9A0413F412

urn:lsid:ipni.org:names:77216565-1

Figs 3 , 4

##### Type.

China. Guangdong: Huizhou, Longmen, on damp slopes of ravines in broad-leaved forests, 115 m, 13 October 2019, *S.Jin Zeng 982* (***holotype***: IBSC!; isotypes: CANT!, KUN!, PE!).

##### Diagnosis.

The new species *Tigridiopalma exalata* resembles *T. magnifica* in having a short stem, huge basal leaves, scorpioid cymes, and 5-merous flowers, but differs in its ribbed and densely pale yellow puberulent petioles, connectives of longer stamens with a distinct dorsal short spur at their base, and wingless capsules.

**Figure 3.**
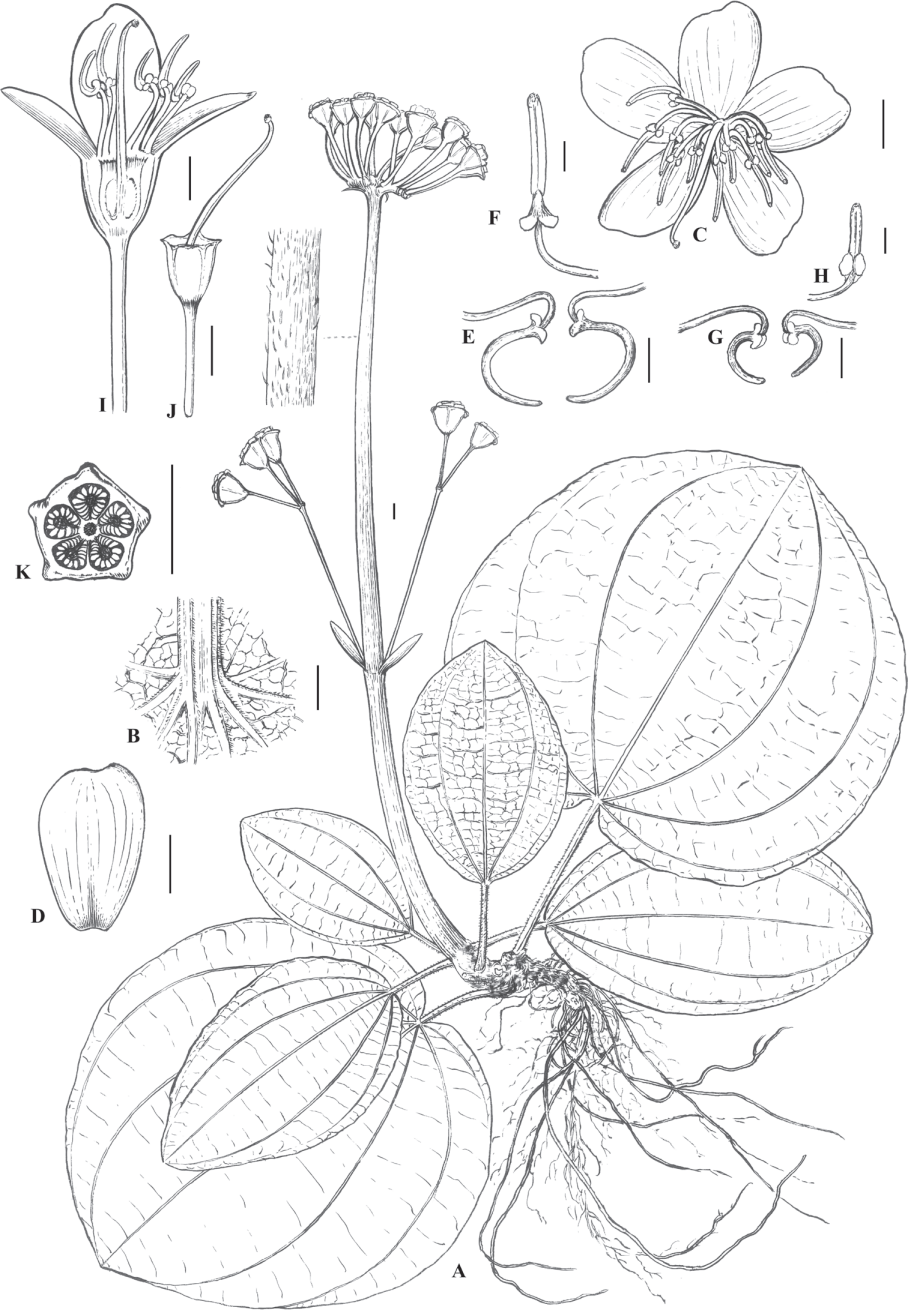
*Tigridiopalma exalata* S.Jin Zeng, Y.C.Xu & D.F.Cui: **A** plant **B** leaf base, abaxial view **C** opening flower, front view **D** petal, front view **E** longer stamens, side view **F** longer stamen, front view **G** short stamens, side view **H** short stamen, front view **I** opening flower, longitudinal section **J** hypanthium, with attached style, longitudinal section. Scale bars: 5 mm. Drawn by Ding-Han Cui.

##### Description.

Perennial herbs, succulent, with raphides in both vegetative and reproductive parts. Stems stoloniferous, 3–5 cm long, internodes indistinct. Leaves in a basal or sub-basal rosette, decussate; petiole somewhat square in cross-section, 6–21 cm long, abaxially ribbed, densely pale yellow puberulent; leaf blade cordate, 16–30(–58) × 16–34(–54) cm, slightly fleshy, base cordate, apex subrounded, margin ciliate and irregular abruptly denticulate, adaxial surface green, glabrous, abaxial surface usually purple, densely pale yellow puberulent on veins; secondary veins 3–4 on each side of the midvein, conspicuous; tertiary veins numerous, parallel, and connecting with secondary veins. Inflorescences terminal, scorpioid cymes, 12–50 flowers; peduncle nearly rounded in cross-section,13–27 cm long, densely pale yellow puberulent; bracts linear, ca. 0.1 cm, puberulent, caducous. Pedicel nearly rounded in cross-section, 1.2–2.0 cm long, puberulent. Hypanthium funnelform to cup-shaped, 5-sided, wingless, ca. 0.6 × 0.6 cm, puberulent, apex truncate. Calyx lobes triangular-semiorbicular, less than 0.1 cm, puberulent, apex apiculate. Petals purple, broadly obovate, ca. 1.0–1.5 × 0.7–1.0 cm, oblique, almost rhomboid, apex white, truncate and oblique. Stamens 10, 5 long antisepalous and 5 short antipetalous, arranged in 2 whorls. Antisepalous (longer) stamens 1.7–2.2 cm long; anthers 0.7–1.0 cm long; connective decurrent, basally with 2 ventral tubercles and a dorsal short spur. Antipetalous (shorter) stamens 1.2–1.5 cm long; anthers 0.6–0.8 cm long; connective slightly decurrent, basally with 2 ventral tubercles and a dorsal short spur. Ovary half-inferior, ovoid, apex with membranous crown; crown 5-lobed, lobe margins ciliate; placentas short stalked. Capsule funnelform cup-shaped, apex truncate, dehiscence poricidal; crown woody, 5-lobed, exserted ca. 0.2 cm beyond calyx, margin irregularly denticulate. Seeds more than 100, light brown, ca. 0.1 cm long.

##### Phenology.

Flowering in October–November, fruiting in January–March.

##### Etymology.

The specific epithet combined from *ex*- (lacking) and *alatus* (winged) which refers to the wingless capsules.

##### Vernacular name.

Hui Zhou Hu Yan Hua (Chinese pronunciation); 惠州虎颜花 (Chinese name).

##### Distribution.

**Figure 4.**
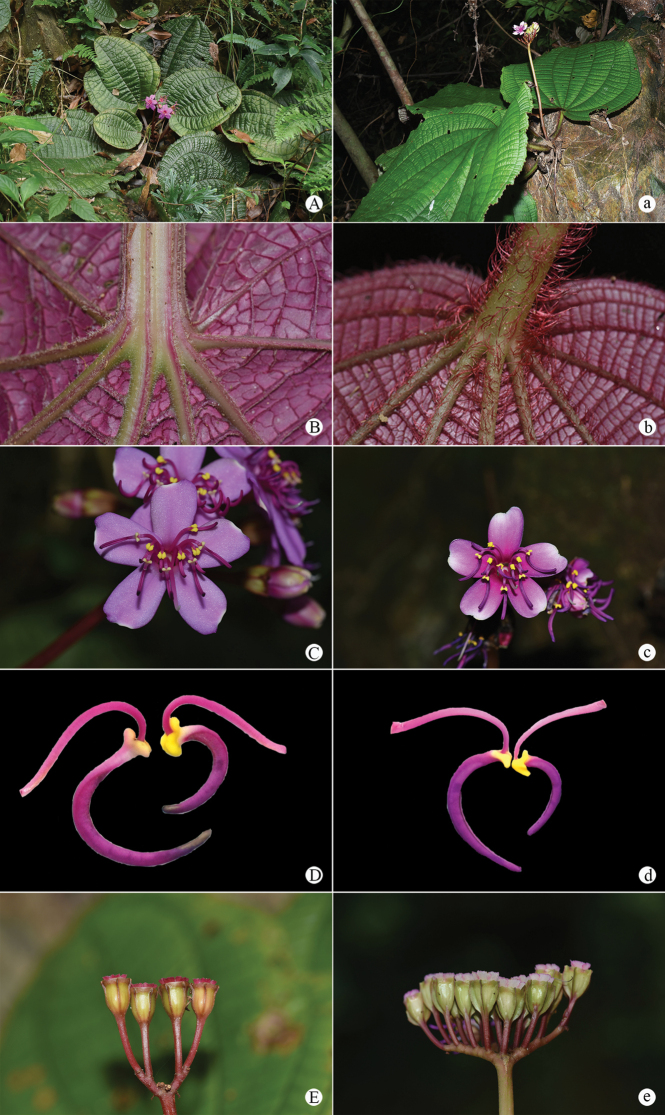
Comparison of *Tigridiopalma exalata* and *T. magnifica*: **A–E***Tigridiopalma exalata***a–e***T. magnifica***A, a** flowering plants **B, b** leaf bases, abaxial view **C, c** flowers, front view **D, d** stamens, side view **E, e** infructescences, side view. Photographed by Si-Jin Zeng.

*Tigridiopalma exalata* grows in damp, shaded but well-drained places in broad-leaved forests, in elevations between 100 m and 350 m in Huizhou, Guangdong Province, China (Fig. 4).

##### Preliminary conservation assessment.

About 1000 mature *Tigridiopalma exalata* individuals from one locality have been found in less than 60 km^2^ up to now. This area can be classified as the extent of occurrence. The plants are not well protected in a Forest Park and the populations are severely fragmented. This species has horticultural potential as an ornamental plant. According to the IUCN Standards and Petitions Committee (2019), a category of Endangered (EN) is recommended for *Tigridiopalma exalata* for the present.

##### Paratype.

China. Guangdong: Huizhou, Longmen, on damp slopes of ravines in broad-leaved forests, 121 m, 25 November 2017, *S.Jin Zeng* 397 (CANT!, IBSC!).

## Supplementary Material

XML Treatment for Tigridiopalma exalata

## Supplementary materials

Tables S1–S3 Data type tables Explanation note **Table S1.** Primers used in this study. **Table S2.** Vouchers and accession numbers of the specimens used in this study. Newly generated sequences are indicated in bold, other sequences of two makers (*ndhF*+*rpl16*) were extracted from published complete chloroplast genomes. **Table S3.** Summary features of sequences used for phylogenetic analysis. PIS, parsimony-informative sites. https://binary.pensoft.net/file/531681Copyright notice: This dataset is made available under the Open Database License (http://opendatacommons.org/licenses/odbl/1.0/). The Open Database License (ODbL) is a license agreement intended to allow users to freely share, modify, and use this Dataset while maintaining this same freedom for others, provided that the original source and author(s) are credited.Si-Jin Zeng, Ye-Chun Xu, Gang-Tao Wang, Peng Jia, Da-Fang CuiM
