# Supplementary material for: Tigridiopalma exalata, a new and endangered species of Melastomataceae from China
Source: PhytoKeys. 2021 Apr 16;176:33–42. doi: 10.3897/phytokeys.176.63619 (PMC8065001; doi:10.3897/phytokeys.176.63619)
Supplement: Tables S1–S3 [file phytokeys-176-033-s001.docx]

**Table S1.** Primers used in this study.

| Primer | Sequence (5’ to 3’) | Reference |
| --- | --- | --- |
| ITS-17SE | ACGAATTCATGGTCCGGTGAAGTGTTCG | Thiv *et al.* 1999 |
| ITS-26SE | GAATTCCCCGGTTCGCTCGCCGTTAC | Thiv *et al.* 1999 |
| *ndhF*-972F | GTCTCAATTGGGTTATATG | Olmstead and Sweere 1994 |
| *ndhF*-1955R | CGATTATATGACCAATCATA | Olmstead and Sweere 1994 |
| *rbcL*-1F | ATGTCACCACAAACAGAAAC | Fay *et al.* 1997 |
| *rbcL*-1352R | CTTCACAAGCAGCAGCTAGGTCAGGACTCC | Wolfe and Pamphilis 1998 |
| *rpl16*-71F | GCTATGCTTAGTGTGTGACTCGTTG | Jordan *et al.* 1996 |
| *rpl16*-1516R | CCCTTCATTCTTCCTCTATGTTG | Kelchner and Clark 1997 |

**Table S2.** Vouchers and accession numbers of the specimens used in this study. Newly generated sequences are indicated in bold, other sequences of two makers (*ndhF*+*rpl16*) were extracted from published complete chloroplast genomes.

| Species | Voucher | ITS | *ndhF* | *rpl16* |
| --- | --- | --- | --- | --- |
| *Allomorphia balansae* | Liu 451 (SYS) | MG644470 | MK994792 | MK994792 |
| *Allomorphia urophylla* | Liu 592 (SYS) | MN031230 | MK994849 | MK994849 |
| *Anerincleistus bracteatus* | Zhou et al. 698 (SYS) | MN031178 | MK994899 | MK994899 |
| *Anerincleistus setulosus* | Zhou et al. 660 (SYS) | MN031185 | MK994881 | MK994881 |
| *Barthea barthei* | Y.C. Cai s.n. (SYS) | MN031159 | MK994907 | MK994907 |
| *Blastus auriculatus* | Liu 542 (SYS) | MG644463 | MK335944 | MK335944 |
| *Blastus cochinchinensis* | Liu 446 (SYS) | MG644465 | MK994909 | MK994909 |
| *Bredia fordii* | Liu 444 (SYS) | MG644400 | MK994790 | MK994790 |
| *Bredia hirsuta* | Liu 634 (SYS) | MN031213 | MK994872 | MK994872 |
| *Bredia longiradiosa* | Liu 486 (SYS) | MN031235 | MK994807 | MK994807 |
| *Cyphotheca montana* | Liu 596 (SYS) | MG644447 | MK994852 | MK994852 |
| *Driessenia glanduligera* | Zhou et al. 657 (SYS) | MN031182 | MK994879 | MK994879 |
| *Driessenia phasmolacuna* | C.W. Lin 659 (SYS) | MN031199 | MK994923 | MK994923 |
| *Fordiophyton faberi* | Liu 588 (SYS) | MN031161 | MK994846 | MK994846 |
| *Fordiophyton strictum* | Liu 514 (SYS) | MN031228 | MK994816 | MK994816 |
| *Heteroblemma serpens* | Liu 671 (SYS) | MN031200 | MK994886 | MK994886 |
| *Medinilla amplectens* | Zhou et al. 663 (SYS) | MN031219 | MK994882 | MK994882 |
| *Medinilla beamanii* | Zhou et al. 658 (SYS) | MN031220 | MK994880 | MK994880 |
| *Medinilla petelotii* | Liu 589 (SYS) | MN031218 | MK994847 | MK994847 |
| *Medinilla speciosa* | Zhou et al. 669 (SYS) | MN031221 | MK994885 | MK994885 |
| *Oxyspora paniculata* | Liu 523 (SYS) | MN031212 | MK994819 | MK994819 |
| *Phyllagathis gigantifolia* | Zhou et al. 659 (SYS) | MN031177 | MK994779 | MK994779 |
| *Phyllagathis gymnantha* | C.W. Lin 625 (TAIF) | MG993334 | MK994918 | MK994918 |
| *Phyllagathis osmantha* | C.W. Lin 567 (TAIF) | MG993336 | MK994915 | MK994915 |
| *Phyllagathis postrata* | C.W. Lin 640 (TAIF) | MG993332 | MK994919 | MK994919 |
| *Phyllagathis rajah* | C.W. Lin 644 (TAIF) | MG993339 | MK994921 | MK994921 |
| *Phyllagathis rotundifolia* | Zhou et al. M50 (SYS) | MG644436 | MK994912 | MK994912 |
| *Phyllagathis rufa* | Zhou et al. 679 (SYS) | MN031209 | MK994890 | MK994890 |
| *Phyllagathis stellata* | C.W. Lin 643 (TAIF) | MG993333 | MK994920 | MK994920 |
| *Plagiopetalum esquirolii* | Liu 594 (SYS) | MN031202 | MK994851 | MK994851 |
| *Plagiopetalum serratum* | Liu 717 (SYS) | MN031170 | MK994902 | MK994902 |
| *Sarcopyramis napalensis* | Liu 628 (SYS) | MN031207 | MK994868 | MK994868 |
| *Scorpiothyrsus oligotrichus* | Liu 454 (SYS) | MG644440 | MK994794 | MK994794 |
| *Scorpiothyrsus shangszeensis* | Liu 626 (SYS) | MN031186 | MK994866 | MK994866 |
| *Sonerila cantonensis* | Liu 449 (SYS) | MG644491 | MK994791 | MK994791 |
| *Sonerila plagiocardia* | Liu 642 (SYS) | MN031191 | MK994876 | MK994876 |
| *Sporoxeia clavicalcarata* | Liu 716 (SYS) | MN031176 | MK994901 | MK994901 |
| *Sporoxeia latifolia* | Liu 524 (SYS) | MN031201 | MK994820 | MK994820 |
| *Styrophyton caudatum* | Liu 615 (SYS) | MN031203 | MK994860 | MK994860 |
| *Tashiroea sinensis* | Liu 569 (SYS) | MG644398 | MK994837 | MK994837 |
| *Tashiroea yaeyamensis* | Liu 631 (SYS) | MN031210 | MK994870 | MK994870 |
| *Tigridiopalma exalata* | Zeng 397 (CANT) | **MW722954** | **MW727281** | **MW727282** |
| *Tigridiopalma magnifica* | Liu 429 (SYS) | MG644449 | MF663760 | MF663760 |
| **outgroup:** |  |  |  |  |
| *Dissochaeta vacillans* | Fan 15703 (SYS) | MG644478 | MK994856 | MK994856 |
| *Pseudodissochaeta lanceata* | Liu 593 (SYS) | MN031232 | MK994850 | MK994850 |

**Table S3** Summary features of sequences used for phylogenetic analysis. PIS, parsimony-informative sites.

| Marker | Accessions sampled | Aligned base pairs | Variable sites | % Variable sites | Conserved sites | PIS | % PIS |
| --- | --- | --- | --- | --- | --- | --- | --- |
| nrITS | 45 | 713 | 380 | 53% | 305 | 278 | 39% |
| *ndhF* | 45 | 951 | 200 | 21% | 703 | 92 | 10% |
| *rpl16* | 45 | 1030 | 188 | 18% | 780 | 83 | 8% |
| *combined* | 45 | 2694 | 768 | 29% | 1788 | 453 | 17% |
